# Digital Triage Tools for Sexually Transmitted Infection Testing Compared With General Practitioners’ Advice: Vignette-Based Qualitative Study With Interviews Among General Practitioners

**DOI:** 10.2196/49221

**Published:** 2024-01-22

**Authors:** Kyma Schnoor, Anke Versluis, Niels H Chavannes, Esther P W A Talboom-Kamp

**Affiliations:** 1 Public Health and Primary Care Leiden University Medical Center Leiden Netherlands; 2 National eHealth Living Lab Leiden University Medical Center Leiden Netherlands; 3 Zuyderland Sittard-Geleen Netherlands

**Keywords:** eHealth, digital triage tool, sexually transmitted infection, STI, human immunodeficiency virus, general practitioners, GPs decision-making, digital health, diagnostic, sexually transmitted disease, STD, sexually transmitted, sexual transmission, triage, artificial intelligence, HIV, diagnostics, diagnosis, vignette, vignettes, interview, interviews, best practice, best practices, thematic analysis, referral, medical advice

## Abstract

**Background:**

Digital triage tools for sexually transmitted infection (STI) testing can potentially be used as a substitute for the triage that general practitioners (GPs) perform to lower their work pressure. The studied tool is based on medical guidelines. The same guidelines support GPs’ decision-making process. However, research has shown that GPs make decisions from a holistic perspective and, therefore, do not always adhere to those guidelines. To have a high-quality digital triage tool that results in an efficient care process, it is important to learn more about GPs’ decision-making process.

**Objective:**

The first objective was to identify whether the advice of the studied digital triage tool aligned with GPs’ daily medical practice. The second objective was to learn which factors influence GPs’ decisions regarding referral for diagnostic testing. In addition, this study provides insights into GPs’ decision-making process.

**Methods:**

A qualitative vignette-based study using semistructured interviews was conducted. In total, 6 vignettes representing patient cases were discussed with the participants (GPs). The participants needed to think aloud whether they would advise an STI test for the patient and why. A thematic analysis was conducted on the transcripts of the interviews. The vignette patient cases were also passed through the digital triage tool, resulting in advice to test or not for an STI. A comparison was made between the advice of the tool and that of the participants.

**Results:**

In total, 10 interviews were conducted. Participants (GPs) had a mean age of 48.30 (SD 11.88) years. For 3 vignettes, the advice of the digital triage tool and of all participants was the same. In those vignettes, the patients’ risk factors were sufficiently clear for the participants to advise the same as the digital tool. For 3 vignettes, the advice of the digital tool differed from that of the participants. Patient-related factors that influenced the participants’ decision-making process were the patient’s anxiety, young age, and willingness to be tested. Participants would test at a lower threshold than the triage tool because of those factors. Sometimes, participants wanted more information than was provided in the vignette or would like to conduct a physical examination. These elements were not part of the digital triage tool.

**Conclusions:**

The advice to conduct a diagnostic STI test differed between a digital triage tool and GPs. The digital triage tool considered only medical guidelines, whereas GPs were open to discussion reasoning from a holistic perspective. The GPs’ decision-making process was influenced by patients’ anxiety, willingness to be tested, and age. On the basis of these results, we believe that the digital triage tool for STI testing could support GPs and even replace consultations in the future. Further research must substantiate how this can be done safely.

## Introduction

### Background

The use of eHealth, health services delivered through the internet or related technologies, is increasing, especially since the COVID-19 pandemic [1,2]. The COVID-19 pandemic has shed light on the crucial role of digitization in health care [2]. An important and promising element of digitization in health care are digital triage tools consisting of a questionnaire for patients to identify the risk of a medical problem. These tools use a digital questionnaire typically administered by a health care professional, and an algorithm based on a medical decision tree generates automatic advice for follow-up, for example, a web-based symptom checker. In this paper, we discuss a digital triage tool that advises whether a specific diagnostic test for a specific combination of symptoms is necessary. This specific digital triage tool is based on Dutch medical guidelines.

Such a digital triage tool for different problems and symptoms could be an efficient and accessible method for citizens with medical questions. In addition, this digital triage tool could possibly lower the workload of general practitioners (GPs) as it can replace the triage that health care professionals would do themselves [3]. However, it is important that triage leads to responsible and appropriate care given the situation. Digital triage tools should not result in “over-triage” or “under-triage” [4]. Overtriage is when a patient is advised to undergo a medical treatment or diagnostic test when they do not have an (urgent) medical problem [4]. Undertriage is when a patient is told that they do not have an (urgent) medical problem when they do, with the advice that a diagnostic test or medical treatment is not necessary [4]. It is important to know whether the digital triage tool for diagnostic tests is in line with daily medical practice to maximize its validity.

In daily practice at GPs’ offices, medical guidelines are used to support their decision-making. GPs following guidelines has been an important research subject into the decision-making process of GPs in dermatology has shown that GPs do not always adhere to medical guidelines [5]. For example, concerns about the patient or the relationship between the GP and the patient were sometimes part of the decision-making process [5]. Furthermore, a meta-synthesis of qualitative studies identified GPs’ attitudes toward and experiences with clinical guidelines [6]. First, this study showed that GPs experience tension between their own experiences and the guidelines they must adhere to as guidelines do not consider personal circumstances. Second, GPs are afraid of missing a patient diagnosis. Third, GPs experience that the guidelines do not always fit with patients’ needs, and therefore, GPs act differently from what the guidelines instruct them to do. Earlier reviews have revealed other factors that play a role in the decision-making process of GPs in referrals for diagnostic tests [7-9]. These are, among others, demographic and nonclinical factors such as patient characteristics (eg, age, sex, and social class [8]). In addition, the patient’s quality of life and wishes are nonclinical factors that influence the decision-making process of the GP [7]. Not all those factors are included in medical guidelines and, consequently, in digital triage. All these factors clearly show that the GP makes decisions from a holistic perspective, which makes it even more interesting and important to critically consider decision-making using digital tools from the perspective of the GP. Regarding diagnostic testing, to our knowledge, our study is the first one that compares the advice of GPs with that of a web-based tool. At the same time, this study identifies what factors influence a GP’s decision-making process for a diagnostic test.

### Objectives

If a digital triage tool is of high quality and the patient is adequately advised, a consultation with the GP could be avoided, resulting in an efficient care process for the patient. The GP can also be supported in the hectic daily workload as the patient uses the tool independently [9]. The first objective of this study was to identify whether the advice of the studied digital triage tool aligned with the daily medical practice of the GP. The second objective was to learn which factors influenced the GP’s decision regarding a referral for diagnostic testing. In addition, this research provides insights into the GP’s decision-making process and whether factors are possibly missing from a digital triage tool. As a starting point, we investigated these research questions for sexually transmitted infection (STI) triage as the medical guidelines are straightforward (eg, clear risk factors and answer categories). Much research has been conducted on digital applications for STI testing, such as websites in which tests can be ordered, with positive feedback from patients about their usability [10]. Moreover, research has shown that a digital triage tool can potentially lower the threshold for STI testing [10] as this problem can be associated with feelings of shame [11]. To answer the research questions, a vignette-based qualitative study was conducted based on different STI-related patient cases [12].

## Methods

### Study Design and Participants

A qualitative vignette study was conducted using semistructured interviews with GPs as participants. Data saturation was expected after 10 interviews [13]. There were no specific exclusion criteria. GPs in training, practicing, or retired (for ≤5 y) could participate. In the interviews, the participants were presented with different patient vignettes (see the *Materials* section for details). After each vignette, the participants were asked about their clinical decision regarding STI diagnostic testing and to describe their thinking and decision-making process. This approach is called the “Think Aloud” method, which allows for a description of how information is structured during a problem-solving task [14]. In addition, it provides rich data for analysis [15].

### Ethical Considerations

This study was declared not to fall within the scope of the Dutch Medical Research Involving Human Subjects Act by the departmental ethics committee of the Leiden University Medical Center (reference 22-3002).

### Materials

A vignette is a short hypothetical description of a patient representing a standardized combination of specific characteristics [16]. Vignettes made it possible to present patients with the same characteristics to every participant (eg, complaints, relationship status, and age) and, in this way, minimize variations between patients, which is not possible in real life. In this study, the vignettes were based on different aspects of the Dutch medical guidelines for STI testing [17]. In the medical guidelines, different aspects are taken into account to calculate the risk of an STI, such as endemic areas, unsafe sex, and different complaints. The following factors were incorporated into the vignettes: age, gender, sexuality, relationship status, employment (eg, full-time job or student), history of unsafe sex and how long ago it took place, number of sexual partners, frequency of unsafe sex, frequent GP visits, symptoms, and ethnicity. Some of these factors are not in the guidelines but were included to research whether they influenced the decision-making process of the GP (eg, situation and if the GP was visited often by that patient). In addition, the vignettes were designed in such a way that they would lead to advice from participants to undergo a diagnostic test for STIs or not. In total, 6 different vignettes were created and used (Multimedia Appendix 1). In Textbox 1, a short description of the vignettes is provided. The Dutch vignettes were designed with a GP and checked by another GP. An example of a translated vignette can be found in Textbox 2.

**Short description of the vignettes.**

**Vignette 1**

- Woman, aged 20 years, from Spain, student, had unsafe sex multiple times >3 weeks ago, itching of the vagina, does not visit her general practitioner (GP) often

**Vignette 2**

- Man, aged 26 years, plumber, steady relationship, has irritation at the urethra and sensitivity when urinating, visits GP often

**Vignette 3**

- Woman, aged 17 years, high school student, had unsafe sex <3 weeks ago with no complaints, the first time she comes to the practice

**Vignette 4**

- Man, aged 24 years, has a relationship with a man, his partner has sexual contact with other men, has difficulty urinating

**Vignette 5**

- Woman, aged 45 years, has a steady relationship but thinks her partner cheated 6 months ago, has contact bleeding, visits the GP often

**Vignette 6**

- Woman, aged 35 years, has a steady relationship, comes from Surinam, has a burning sensation when urinating, visits her GP often

**Vignette 1 translated from Dutch to English.**

- Mrs A is aged 20 years and studies in the Netherlands but comes from Spain originally. She has not visited you at the practice often. She is not in a committed relationship and has had unprotected sex several times in the past 6 months for more than 3 weeks. She experiences vaginal discharge and itching and irritation in her vagina. She wonders whether she might have a sexually transmitted infection.

### Procedure

Participants were recruited via a LinkedIn post that included the email address of the researcher. Interested participants were instructed to send an email if they wanted to take part. In addition, participants were emailed from the network of the researchers, and the GPs could reply to the email if they wanted to participate. Interested participants were sent information and the informed consent form. In addition, different data and time points were included in the interviews, which could be face-to-face or digital (based on the preference of the participant). Participants had the right to withdraw at any time.

An interview protocol guided the semistructured interviews (Multimedia Appendix 2). All interviews were audio recorded. Each interview started with a short explanation of the study. The first vignette was then read out loud to the participant. They were asked whether they would advise undergoing diagnostic tests for STIs. Next, they were asked to share their reasoning process. These 2 steps were repeated for each vignette (ie, 6 in total). The first interviews were conducted with both interviewers present (KS and Fleur Rekveld), and KS was the lead. The other interviews were conducted by KS, Fleur Rekveld, or both.

### Service: Digital Triage Tool

The digital triage tool was developed by a Dutch diagnostic center [18] based on a decision tree with Dutch medical guidelines [17]. The digital triage tool was developed in cocreation with GPs and clinical chemists. A Dutch academic knowledge center assessed the digital triage [19]. During triage, users first go through a series of questions. Their answers determine what question they have to answer next and, in the end, what advice is given. For example, the first question is “Did you have unsafe sex?” If the answer is “no,” the advice is not to be tested. If the answer is “yes,” a follow-up question appears: *what is your gender?* Gender is asked about as differences in gender result in different advice (eg, for women users who are advised to undergo a chlamydia test, it means that the service could advise doing a vaginal swab). Ultimately, the digital triage tool advises whether a diagnostic test for STIs is necessary and, if yes, which one (eg, chlamydia, gonorrhea, or HIV). The digital triage tool is now used in 2 digital services of the diagnostic company where patients can order diagnostic tests themselves with or without a health care professional. These diagnostic services are Directlab, where users can order web-based diagnostic test packages independent of a health care professional, and Homelab, where patients in the digital environment of their GP can order diagnostic test packages. In regular daily practice in the Netherlands, the patient needs to ask for a consultation with the GP (on the phone or in person) and ask for a diagnostic test for STIs. In this situation, the GP performs triage to identify whether it is necessary to conduct an STI test.

### Data Analysis

To determine the diagnostic test advice of the digital triage tool, the characteristics of each vignette were entered into it. The ensuing advice was compared with the test advice of the GPs per vignette. To learn which factors influenced the GPs’ decision-making process, the combination of the think-aloud process, vignettes, and semistructured interviews was used as a triangulation method to obtain a complete range of data to result in a strong conclusion [12,20]. All interviews were transcribed (intelligent) verbatim. When the transcripts were completed and uploaded to ATLAS.ti (version 22; ATLAS.ti Scientific Software Development GmbH), the audio recordings were deleted. In total, 2 authors (Fleur Rekveld and KS) conducted the qualitative data analysis according to the principles of thematic analysis. Fleur Rekveld and KS developed a preliminary coding scheme based on the coded data from the first 8 participants. The final coding scheme emerged after all the coding was performed by the 2 authors independently. The codes were grouped into themes and subthemes.

## Results

### Characteristics of the Study Population

Data saturation was reached after 10 interviews. The characteristics of the participants are presented in Table 1. Their ages ranged from 32 to 70 years, with a mean of 48.30 (SD 11.88) years. The number of men and women was almost equal (6/10, 60% and 4/10, 40%, respectively). Of the 10 GPs, 1 (10%) was retired, 3 (30%) were working part time as GPs, and 6 (60%) were working full time.

**Table 1:** Characteristics of the participants.

Participant	Age (y)	Gender	Employment status
1	32	Woman	Part time
2	55	Man	Full time
3	38	Man	Part time
4	59	Man	Full time
5	70	Man	Retired
6	53	Man	Full time
7	55	Woman	Full time
8	43	Man	Full time
9	38	Woman	Part time
10	40	Woman	Full time

### Testing Advice of Digital Triage Tool Versus GPs

Table 2 shows, for each vignette, whether the digital tool would advise conducting an STI test and what each participant would advise to do. For 50% (3/6) of the vignettes (ie, numbers 1, 4, and 5), the digital triage tool’s advice aligned with all participants’ advice. For all 3 vignettes, the advice was to conduct a diagnostic test for STIs. For those 3 vignettes, the patients’ risk factors were sufficiently clear for the participants to advise to conduct a test.

In vignette 1, the most important decision-making factor was the patient’s age; young age combined with women was an important factor influencing the participants’ test advice as having an STI could make this woman infertile. Participant 7 answered the following:

I would test her, always with women of her age who are sexually active.

In addition, unsafe sex was an important factor in the decision to test.

For vignette 4, the main factor in advising to test was the “men having sex with men” risk factor. Participant 5 answered the following:

It is male-male contact, and in addition, there are changes in sexual contacts so that he can do an STI test.

For vignette 5, all participants would advise conducting an STI test as well. Furthermore, 80% (8/10) mentioned that they would also conduct cervical cancer diagnostic tests because of the symptom of contact bleeding. Participant 9 mentioned the following:

In the case of contact bleeding, more research than only an STI is needed. It could be Chlamydia, but a smear test is needed to exclude cervical cancer.

For the other 50% (3/6) of the vignettes, not all participants gave the same advice as each other or as the digital triage tool. For vignette 2, a total of 60% (6/10) of the participants agreed with the advice of the digital tool, and for vignettes 3 and 6, the proportions were 70% (7/10) and 80% (8/10), respectively. It is important to mention that the initial answer of the participants is presented in Table 2. It could be the case that participants answered “no” to advising an STI test for the patient initially. However, the participants mentioned that they would advise conducting an STI test after excluding other diseases. In addition, sometimes, the participants wanted more information about the patient’s situation before advising to conduct an STI test.

For vignette 2, most participants wanted to know more about the patient’s case before giving the advice to test for an STI. In addition, they wanted to conduct a physical examination or other tests, such as a test to exclude urinary infection, as the patient’s symptoms seemed not totally compliant with those of an STI. Participant 2 said the following:

I would like to know a little more; why does he think he has an STI? Does he have other contacts next to his current relationship or an open relationship? Has he heard anything from his wife?

Participant 4 answered the following:

I would check his urine.

Participants answered that the symptoms and risk factors were too unclear to advise an STI test. A minority of the participants would test for an STI to exclude it or to satisfy the patient’s request. Participant 2 answered the following:

He asked for an STI test so I would do one.

The participants mentioned that, sometimes, a patient does not have an apparent reason for wanting to take an STI test or the patient has no symptoms that fit with those of an STI. However, sometimes patients do not want to discuss this in detail, and participants found it important to allow for testing at a low threshold if patients asked for it themselves. Participant 9 mentioned the following:

Maybe he (or his wife) is cheating, and they do not want to tell you that directly...It is always the question if the patient is honest with you, so I would test at a low threshold after I did a urine infection test, and then I think he would accept that.

**Table 2:** Advice of the digital tool and the participants to test for a sexually transmitted infection.

	Digital triage tool	P^a^1	P2	P3	P4	P5	P6	P7	P8	P9	P10	Agreement, n (%)^b^
Vignette 1	Yes	Yes	Yes	Yes	Yes	Yes	Yes	Yes	Yes	Yes	Yes	10 (100)
Vignette 2	No	No	Yes	No	No	Yes	No	No	Yes	No	Yes	6 (60)
Vignette 3	Later	Later	Later	Later	Later	No	Yes	Yes	Later	Later	Later	7 (70)
Vignette 4	Yes	Yes	Yes	Yes	Yes	Yes	Yes	Yes	Yes	Yes	Yes	10 (100)
Vignette 5	Yes	Yes	Yes	Yes	Yes	Yes	Yes	Yes	Yes	Yes	Yes	10 (100)
Vignette 6	Yes	No	Yes	Yes	Yes	Yes	Yes	Yes	Yes	No	Yes	8 (80)

^a^P: participant.

^b^Percentage of participants who agreed with the advice of the digital triage tool.

For vignette 3, most participants (7/10, 70%) answered that the patient could take an STI diagnostic test but at a later time. At this time, it was too early to detect an STI. A total of 20% (2/10) of the participants also mentioned that they would talk to the patient about her contraception and provide education about safe sex. Participant 2 said the following:

She had unsafe sex, so I would do two things. Maybe check if she uses birth control, and I would tell her that she can do an STI test after two weeks.

Vignette 6 involved a patient from an endemic area. In total, 25% (2/8) of the participants who agreed with the advice of the digital tool mentioned the endemic area as a reason for testing. Participant 10 mentioned the following:

I would ask her some more questions; however, she is from Surinam, a risk area. So I would test her at a low threshold, especially for a serological test.

The other 62% (6/8) of the participants mentioned low-threshold testing because of the patient’s symptoms. Most participants (6/10, 60%) mentioned that they would check for a urinary infection, some before conducting an STI test and others in addition to it. Participant 1 mentioned the following:

I would check her urine first to ensure she has no urinary infection.

It is important to note that almost all participants mentioned that, if a patient requested an STI test, they would meet the request. They also mentioned that, in some cases, they would also give patients more information about safe sex or conduct a physical examination. The decision to do so often depended on age or other risk factors such as contact bleeding. Especially in the case of younger patients, GPs educated them about safe sex and birth control. However, this information provision was not part of their decision-making process but rather of their consultation.

### Extra Factors That Influenced the Decision of the GPs

There were several factors that the participants considered in their decision that were not included in the digital triage tool. The most important additional patient-related factors were anxiety about infection, the wishes of the patient, and age. Among all participants (10/10, 100%), the patient’s anxiety was an additional reason for referring them to an STI test. The participants reasoned that a request for an STI test is not made easily and that there may be an unknown reason behind it. In their opinion, when patients experience fear-related stress, it might harm their health. Participant 10 mentioned the following:

Sometimes you feel that there is more than they want to say, and then you decide to test at a low threshold.

Age played a role in the decision-making process of the GPs. This was especially the case in vignettes 1 and 3. The GPs mentioned that checking for STIs was important at a fertile age, especially for women. In the Dutch medical guidelines, it is noted that, below the age of 25 years, there needs to be a low threshold for STI testing even if patients report no complaints. Participant 6 answered the following in the interview about vignette 3:

Especially in younger patients, you want to know what they know about sex and the transmission of STIs.

In 2 vignettes, the GPs felt the need to ask additional questions or conduct a physical examination. The digital triage tool only provides advice on an STI test. However, the symptoms may also indicate a urinary tract infection or a stage of cervical cancer. These tests are not advised via the digital tool but were advised by the participants in this study for those 2 vignettes.

One GP also considered who had to pay for the test and whether it was affordable. Participant 3 mentioned the role of the payer or possible reimbursement in the decision. He answered the following about vignette 6:

If she wants to pay for a test and she wants to do a test...Then, she can do a test.

In summary, it can be generally said that GPs in this study paid extra attention to patient-related factors such as fear of infection, desire to undergo the test, and young age when deciding whether to request an STI test.

## Discussion

### Principal Findings

In this study, we tried to identify whether the advice of a digital triage tool based on medical guidelines aligned with GPs’ medical practice. The results showed that other factors, which are not part of the guidelines, played a role in the GPs’ decision-making process when determining whether to advise an STI test for a patient. The most important additional patient-related factors were the patient’s anxiety, wishes, and age. The GPs also considered who had to pay for the test and whether it was affordable. Finally, the GPs were willing in some vignettes to ask additional questions or conduct a physical examination. The most notable factors are discussed in this section and compared with the literature.

In line with other research, the GPs’ decision to test depends sometimes on the anxiety and wishes of the patient [7]; these factors were not included in the studied digital triage tool. This additional aspect aligns with the research by Hajjaj et al [5,7]. In addition, our results align with those of a study that researched the barriers to following guidelines among GPs [6] that showed that the patient’s preferences were considered more important than following guidelines.

The interviews showed that the age of the patients was an important factor that influenced the GPs’ advice. Specifically, younger age was an important reason to advise an STI test because of the risk of infertility and the sexual activity in this group. Age was not included as a factor in the digital triage tool. As STIs mainly occur under the age of 30 years, it is not surprising that GPs tend to advise testing more for patients in this age group [21].

From the literature, it was found that the factor “knowing the patient” influences the decision-making process of GPs [22]. Accumulated knowledge about the patient influences the context and interpretation of the conversation between the patient and the health care professional, especially in the case of psychosocial or unspecific problems such as fatigue. However, in this study, knowing the patient was not a factor that was considered in the vignettes. For this reason, the decisions that the GPs made in this study could be different in real life as they might know the patients.

In addition to patient-related factors (eg, the wishes of the patient), GP-related factors also influenced the decision-making process. The extent to which GPs were open to discussion with patients about why they wanted an STI test or to which GPs were willing to address patients’ concerns influenced the decision. In addition, based on the findings of this study, it seems that the GPs expressed a preference for obtaining a complete set of information before deciding. For example, some GPs wanted to have more information about the situation of the patients and their partners. In some cases, GPs wanted to conduct a physical examination or other diagnostic tests (eg, urinary infection) to exclude other diseases. The digital triage tool is strictly bound to the guidelines set up without paying attention to, for example, the anxiety of the patient or the need for additional information. Other guidelines have been developed for possible symptoms of urinary tract infection or cervical problems, which have not yet been combined on the internet.

The advice of the digital triage tool is straightforward and always in line with a strict algorithm. In this study, GPs were found to recommend a diagnostic test for STIs more often than the digital tool. In the Netherlands, a study showed that unnecessary diagnostics (overdiagnostics) are a common problem among Dutch GPs; slightly more than half of the participating GPs indicated that patients could submit a complaint for not requesting an examination that was indicated and that this played a role to some or a significant extent in the request for diagnostic testing [23].

Our study did not investigate whether the digital tool can prevent overdiagnostics, but we assume that it can be a powerful decision support tool for daily general practice, just as tools for pharmacotherapy are already in use. More research is needed to confirm this.

Another possible reason why GPs are more inclined to test seems to be that it could save them time [24]. For example, if a patient has vague symptoms, it would be easy to request some tests first without having a thorough conversation. Another possible reason specifically for low-threshold STI testing could be feelings of embarrassment to ask about sexual behavior [25]. Recently, a Dutch center for sexual health found that talking about sexual behavior is not done as often as it should by health care professionals [26]. This could be seen as an additional justification for supporting GPs with digital tools for STI testing.

This study does not suggest that digital triage is the holy grail to prevent overdiagnostics or that it is *the* solution to lower the work pressure of GPs. However, this vignette study confirms that GPs have a more holistic approach to their patients compared with a digital triage tool. A digital triage tool primarily relies on specific responses to predefined questions, whereas a GP can consider more factors such as social factors, lifestyle, and personal context. On the one hand, the comprehensive perspective of GPs might result in a higher frequency of diagnostics when compared with a digital triage tool. This is due to the GPs considering additional factors. Given the high workload and time constraints of GPs, the investigated digital tool can play a helpful role in daily decision-making. In contrast, this holistic approach by GPs could potentially lead to fewer diagnostics. Given their deep understanding of the patients’ condition, GPs are better positioned to assess the necessity of tests.

This study has several limitations. It could be that social desirability influenced the GPs’ answers on the vignettes and interviews. Potentially, the advice of the GPs was more in line with the guidelines compared with that in their daily practice as they were aware of the fact that they were part of research on this topic [12]. It is also worth mentioning that there could be a disparity between what people think they would do in a particular situation and their actual behavior [27]. In addition, this study is not generalizable to the entire field of diagnostics at general practices because of its focus on STI testing. As a starting point, this study identified factors that influenced the decision-making process of GPs for STI testing. In future research, we recommend investigating digital tools and the decision-making process of GPs for other common diagnostic tests.

A strength of this study is the combination of the vignette method, the think-aloud process, and the semistructured interviews, which aimed to obtain a complete range of data on the topic (triangulation). Although no actual patients were included in this study, we aimed to make the vignettes as valid as possible by developing and testing them with GPs. In addition, providing the same vignettes to different GPs made it easier to compare patients within different general practices instead of comparing real-life patients with different complaints and characteristics. Currently, we are working on a real-life study in which patients in the waiting room of a GP’s office complete digital triage for STI testing (the result of the digital triage tool is not shown to the patient), after which they go on to have their planned consultation with the GP. At this consultation, the GP will also advise whether to test for an STI; the advice of the digital tool and of the GP will be compared. We expect more detailed and practical information to further refine this working method using a digital tool.

A qualitative study in which GPs were interviewed about their general attitude toward the use of digital tools by patients in their practice showed that GPs’ attitudes toward digital STI diagnostic services were positive, and they acknowledged that the use of eHealth in their practice could result in a more efficient workflow [28].

It will be interesting to further investigate whether GPs are also willing to use digital triage tools as a standard gateway for their practice for some diagnostic tests. When a digital triage tool is implemented and integrated into the care pathway, it is important to investigate what users think of this integration and whether they are satisfied with this change in their way of working. For future research, it could be beneficial to make a comparison of the experiences of patients with a digital triage tool, triage at the GP’s office, and a mix. Notably, recent studies on digital chatbots for medical questions have shown that patients perceived the chatbot’s responses to be superior to those provided by GPs [29]. For future applications, it is essential to consider patients’ eHealth literacy before using a digital triage tool as the primary tool in daily general practice [30,31]; hybrid care might be a solution to address all types of patients. Finally, it is important to realize that the tool in the care pathway needs to stay up-to-date and needs to be changed when the medical guidelines are updated [32]. This study showed that (holistic) factors that are not part of the digital triage tool affect GPs’ decision-making. This is an interesting topic for future research as digital tools and artificial intelligence are increasingly being used in health care. Nowadays, GPs use digital medication prescription tools to support their decision-making, which could help with handwriting errors but also with poor treatment decisions [33]. Another example is an artificial intelligence system that could help GPs decide on the early detection of skin cancer [34,35]. Digital technologies such as these should be researched carefully to see what the impact and consequences are for both GPs and patients.

### Conclusions

This study shows that, in some cases, patients receive different advice to undergo an STI test from a digital tool and from a GP. Other factors that are not part of medical guidelines play a role in the GPs’ decision-making process when deciding whether to request an STI test. The most important additional patient-related factors were the patient’s anxiety, wishes, and age. One GP also considered who had to pay for the test and whether it was affordable. Finally, some GPs expressed a desire to ask additional questions or conduct a physical examination in certain vignettes. In comparison, the digital triage tool adhered more closely to the medical guidelines, with GPs being more inclined than the digital tool to recommend an STI test for the same patient case. Alignment between the digital tool and GP advice only occurred when the risk factors for STI testing were unequivocally evident. This confirms that GPs decide from a holistic perspective. On the basis of these initial findings, we cautiously posit that a digital triage tool for STI testing can potentially support GPs and may even serve as a substitute for in-person consultations in the future. However, it is imperative to conduct further research to establish safe and effective methods for implementing such a transition.

These conclusions should be approached carefully, recognizing that this study represents an initial exploration and that additional research is required to substantiate and refine these findings.

## Appendix

Multimedia Appendix 1 Translated vignettes from Dutch to English.

Multimedia Appendix 2 Semistructured interview protocol.

## Abbreviations

GP: general practitioner
STI: sexually transmitted infection

## References

[1] Eysenbach G (2001). What is e-health?. J Med Internet Res.

[2] Fang ML, Walker M, Wong KL, Sixsmith J, Remund L, Sixsmith A (2022). Future of digital health and community care: exploring intended positive impacts and unintended negative consequences of COVID-19. Healthc Manage Forum.

[3] Gottliebsen K, Petersson G (2020). Limited evidence of benefits of patient operated intelligent primary care triage tools: findings of a literature review. BMJ Health Care Inform.

[4] Schmieding ML, Kopka M, Schmidt K, Schulz-Niethammer S, Balzer F, Feufel MA (2022). Triage accuracy of symptom checker apps: 5-year follow-up evaluation. J Med Internet Res.

[5] Hajjaj FM, Salek MS, Basra MK, Finlay AY (2010). Nonclinical influences, beyond diagnosis and severity, on clinical decision making in dermatology: understanding the gap between guidelines and practice. Br J Dermatol.

[6] Carlsen B, Glenton C, Pope C (2007). Thou shalt versus thou shalt not: a meta-synthesis of GPs' attitudes to clinical practice guidelines. Br J Gen Pract.

[7] Hajjaj FM, Salek MS, Basra MK, Finlay AY (2010). Non-clinical influences on clinical decision-making: a major challenge to evidence-based practice. J R Soc Med.

[8] O'Donnell CA (2000). Variation in GP referral rates: what can we learn from the literature?. Fam Pract.

[9] Duddy C, Wong G (2021). Efficiency over thoroughness in laboratory testing decision making in primary care: findings from a realist review. BJGP Open.

[10] Versluis A, Schnoor K, Chavannes NH, Talboom-Kamp EP (2022). Direct access for patients to diagnostic testing and results using eHealth: systematic review on ehealth and diagnostics. J Med Internet Res.

[11] Balfe M, Brugha R (2010). Disclosure of STI testing activities by young adults: the influence of emotions and social networks. Sociol Health Illn.

[12] Daemers DO, van Limbeek EB, Wijnen HA, Nieuwenhuijze MJ, de Vries RG (2017). Factors influencing the clinical decision-making of midwives: a qualitative study. BMC Pregnancy Childbirth.

[13] Hennink M, Kaiser BN (2022). Sample sizes for saturation in qualitative research: a systematic review of empirical tests. Soc Sci Med.

[14] Fonteyn ME, Kuipers B, Grobe SJ (2016). A description of think aloud method and protocol analysis. Qual Health Res.

[15] Lundgrén-Laine H, Salanterä S (2010). Think-aloud technique and protocol analysis in clinical decision-making research. Qual Health Res.

[16] Atzmüller C, Steiner PM (2010). Experimental vignette studies in survey research. Methodol.

[17] Van Bergen J, Dekker J, Boeke A, Kronenberg E, Van der Spruit R, Burgers J (2013). NHG-Standaard Het soa-consult (eerste herziening). Huisarts Wet.

[18] Home page. Saltro.

[19] Home page. National eHealth Living Lab.

[20] Noble H, Heale R (2019). Triangulation in research, with examples. Evid Based Nurs.

[21] Zorg V (2017). SOA: Leeftijd & Geslacht. VZinfo.

[22] Hjortdahl P (1992). The influence of general practitioners' knowledge about their patients on the clinical decision-making process. Scand J Prim Health Care.

[23] Wammes J, Verhoef L, Westert G, Assendelft P, Jeurissen P, Faber M (2013). Onnodige zorg in de Nederlandse gezondheidszorg, gezien vanuit het perspectief van de huisarts. Celsus Academie voor Betaalbare Zorg.

[24] Vervloet M, Bomhoff M, Schellevis F, van Dijk L (2015). Niet te veel en niet te weinig: de balans tussen nodige en onnodige zorg in de huisartsenpraktijk. NIVEL.

[25] Hinchliff S, Gott M, Galena E (2004). GPs' perceptions of the gender-related barriers to discussing sexual health in consultations--a qualitative study. Eur J Gen Pract.

[26] Zuil W Rutgers: seksualiteit onvoldoende besproken binnen de zorg. Skipr.

[27] Erfanian F, Roudsari RL, Heydari A, Bahmani MN (2020). A narrative on using vignettes: its advantages and drawbacks. J Midwifery Reproductive Health.

[28] de Wilt T, Versluis A, Goedhart A, Talboom-Kamp E, van Delft S (2020). General practitioners attitude towards the use of eHealth and online testing in primary care. Clin eHealth.

[29] Ayers JW, Poliak A, Dredze M, Leas EC, Zhu Z, Kelley JB, Faix DJ, Goodman AM, Longhurst CA, Hogarth M, Smith DM (2023). Comparing physician and artificial intelligence chatbot responses to patient questions posted to a public social media forum. JAMA Intern Med.

[30] van der Kleij RM, Kasteleyn MJ, Meijer E, Bonten TN, Houwink EJ, Teichert M, van Luenen S, Vedanthan R, Evers A, Car J, Pinnock H, Chavannes NH (2019). SERIES: eHealth in primary care. Part 1: concepts, conditions and challenges. Eur J Gen Pract.

[31] Heijsters F, van Loon G, Santema J, Mullender M, Bouman M, de Bruijne M, van Nassau F (2023). A usability evaluation of the perceived user friendliness, accessibility, and inclusiveness of a personalized digital care pathway tool. Int J Med Inform.

[32] Stahl L, Spatz M (2003). Quality assurance in eHealth for consumers. J Consum Health Internet.

[33] Armando LG, Miglio G, de Cosmo P, Cena C (2023). Clinical decision support systems to improve drug prescription and therapy optimisation in clinical practice: a scoping review. BMJ Health Care Inform.

[34] Micocci M, Borsci S, Thakerar V, Walne S, Manshadi Y, Edridge F, Mullarkey D, Buckle P, Hanna GB (2021). Attitudes towards trusting artificial intelligence insights and factors to prevent the passive adherence of GPs: a pilot study. J Clin Med.

[35] Sangers TE, Wakkee M, Moolenburgh FJ, Nijsten T, Lugtenberg M (2023). Towards successful implementation of artificial intelligence in skin cancer care: a qualitative study exploring the views of dermatologists and general practitioners. Arch Dermatol Res.

