# Supplementary material for: Digital Triage Tools for Sexually Transmitted Infection Testing Compared With General Practitioners’ Advice: Vignette-Based Qualitative Study With Interviews Among General Practitioners
Source: JMIR Hum Factors. 2024 Jan 22;11:e49221. doi: 10.2196/49221 (PMC10845018; doi:10.2196/49221)
Supplement: Multimedia Appendix 1 [file humanfactors_v11i1e49221_app1.docx]

**Appendix 1.** Translated vignettes from Dutch to English

Vignette 1:

Mrs A is 20 years old, and studies here in the Netherlands but comes from Spain originally. She has not often visited you at the practice. She is not in a committed relationship and has had unprotected sex several times in the past 6 months for more than 3 weeks. She suffers a lot from itching, other discharge and irritation of her vagina. She wonders if she might have an STI.

Vignette 2:

Mr B is 26 years old, is a plumber and has been in a steady relationship with a woman for a few years. He has complaints such as irritation at the urethra and sensitivity when urinating. He wonders what this could be. Could it be an STI? You know Mr B well because he often comes to you with such questions.

Vignette 3:

Mrs C is 17 years old and is coming to your general practice for the first time. She is in her senior secondary school year. Last week she had unprotected sex with a boy. She has no complaints yet, but would still like to do an STI test.

Vignette 4:

Mr D is 24 years old, and a high school teacher. He is in a steady relationship with a man. His husband also has sexual contact with other men. Mr D wants to have a test done to be sure because he sometimes has a difficult time urinating. Furthermore, he does not often visit you at the practice for other matters.

Vignette 5:

Mrs E, aged 45, regularly visits you. She has been in a steady relationship with a man for 2 years now. She has no children and lives alone. She found out that her husband cheated six months ago. She suffers from contact bleeding and therefore wants to have a test done.

Vignette 6:

Mrs F is 35 years old and has a steady relationship with a man. She is from Surinam. She has two children who still live at home. She often visits you at the practice. Occasionally she has a burning sensation when urinating and so she wants to have a test done just to be sure.
