# Supplementary material for: Digital Triage Tools for Sexually Transmitted Infection Testing Compared With General Practitioners’ Advice: Vignette-Based Qualitative Study With Interviews Among General Practitioners
Source: JMIR Hum Factors. 2024 Jan 22;11:e49221. doi: 10.2196/49221 (PMC10845018; doi:10.2196/49221)
Supplement: Multimedia Appendix 2 [file humanfactors_v11i1e49221_app2.docx]

**Appendix 2. Interview protocol**

- 1. **1. Introduction, explantion, informed consent**

1. Welcome, introduction of facilitator
2. Introduction to the topic
3. Explanation of what we are going to do
4. Informed Consent form
5. Practical questions?
6. Demographic questions for the general practitioner
   1. What is your birth year?
   2. Are you still a full-time general practitioner (and how long)?

*questions per vignette*

**2. Questions**

a) You need to make a decision regarding the care for this patient. What would you do for this patient? (If no clear answer: What you do a diagnostic test for sexual transmitted infections?)

b) Why would you do this?

a. What factors do you take into consideration?

b. What is the role of patient characteristics and how they present in your decision?

*Provide examples of characteristics if necessary, like age or how often they see the patient*

c. What are your thoughts about the patient?

d. What do you pay attention to in such patient?

c) Are there any specific things that we did not have discussed yet, but are for you crucial in the decision-making process for the patient?
